# Subtoxic levels hydrogen peroxide-induced production of interleukin-6 by retinal pigment epithelial cells

**Published:** 2010-09-12

**Authors:** Wen-Chuan Wu, Dan-Ning Hu, Hua-Xin Gao, Min Chen, Dawei Wang, Richard Rosen, Steven A. McCormick

**Affiliations:** 1 Department of Ophthalmology, Kaoshiung Medical University Hospital, Kaoshiung, Taiwan; 2 Department of Ophthalmology, College of Medicine, Kaoshiung Medical University, Kaoshiung, Taiwan; 3 Tissue Culture Center, Departments of Pathology and Ophthalmology, New York Eye and Ear Infirmary, New York Medical College, New York, NY; 4 Albert Einstein College of Medicine, Bronx, NY; 5 Office of Chief Medical Examiner of New York City, New York, NY

## Abstract

**Purpose:**

To study the effect of subtoxic levels of hydrogen peroxide (H_2_O_2_) on the expression and release of interleukin-6 (IL-6) by cultured retinal pigment epithelial (RPE) cells and to explore the relevant signal pathways.

**Methods:**

Cultured human RPE cells were stimulated with various subtoxic concentrations of H_2_O_2_ for different periods. Conditioned medium and cells were collected. IL-6 in the medium and IL-6 mRNA in the collected cells were measured using an IL-6 enzyme-linked immunosorbent assay kit and reverse transcriptase polymerase chain reaction, respectively. Nuclear factor-kappaB (NF-κB) in nuclear extracts and phosphorylated p38 mitogen-activated protein kinase (MAPK), extracellular signal-regulated kinase (ERK), and c-Jun N-terminal kinases (JNK) in cells cultured with and without H_2_O_2_ were measured by NF-κB and MAPK enzyme-linked immunosorbent assay kits. Inhibitors of p38 (SB203580), ERK (UO1026), JNK (SP600125), and NF-κB (BAY11–7082) were added to the cultures before the addition of H_2_O_2_ to test their effects_._

**Results:**

Subtoxic levels of H_2_O_2_ (100 µM and less) increased the IL-6 mRNA level and the release of IL-6 protein by the cultured human RPE cells in a dose- and time-dependent manner. This was accompanied by an increase of NF-κB in nuclear extracts and phosphorylated p38 MAPK, ERK, and JNK in cell lysates, particularly in the p38 and NF-κB. The NF-κB inhibitor decreased the H_2_O_2_-induced expression of IL-6. The p38 inhibitor, but not the ERK or JNK inhibitor, completely abolished H_2_O_2_-induced expression of IL-6 by RPE cells. The p38 inhibitor also abolished the increase of NF-κB in nuclear extracts in cells treated with H_2_O_2_.

**Conclusions:**

H_2_O_2_ stimulated the production of IL-6, a key factor in the modulation of immune responses, inflammatory processes, and the occurrence of autoimmune diseases, which recently has been documented to be increased in age-related macular degeneration (AMD). This may be a molecular linkage for the oxidative stress and inflammatory/autoimmune reactions in AMD and may provide a novel target for the treatment of AMD.

## Introduction

Age-related macular degeneration (AMD) is the leading cause of blindness among elderly persons in Western countries [1]. Oxidative stress has been implicated in the pathogenesis of AMD. Reactive oxygen species (ROS) generated from phagocytosis, lipid peroxidation, and photic stress, together with the high oxygen tension in the choroid and in the macular region, contribute to the particular susceptibility to oxidative stress demonstrated in retinal pigment epithelial (RPE) cells in the macular region [1-5].

ROS have two different effects on the cells. Traditionally, they are thought to have cytotoxic effects and are implicated in causing cell death; however, recent studies also suggest that at subtoxic levels, they may influence signaling pathways and play a major role in various aspects of cell function [6-9].

There has been increasing evidence suggesting a role for inflammation, aberrant complement activation, and autoimmune responses in the pathogenesis of AMD [10-26]. It is therefore important to explore mechanisms involved in ROS-induced inflammatory and autoimmune responses.

Interleukin-6 (IL-6) is a pro-inflammatory cytokine. It amplifies immune and inflammatory responses and plays a critical role in the occurrence of autoimmune diseases [27-30]. Elevated IL-6 levels have been observed in various autoimmune diseases, including uveitis [31-33]. Recently, it was reported that serum IL-6 levels correlate with the progression of AMD and high levels of serum IL-6 were associated with the geographic atrophy type of AMD [13,14].

Human RPE cells constitutively express and release IL-6 at a relatively low level [34-38]. Subtoxic levels of hydrogen peroxide (H_2_O_2_) stimulate the production of IL-6 in several cell types [39-43]. However, the effect of H_2_O_2_ on the production of IL-6 by RPE cells has not yet been reported.

We hypothesized that subtoxic levels of H_2_O_2_ may stimulate the production of IL-6 by RPE cells, leading to the stimulation of inflammatory and autoimmune reactions. They may also play a role in the pathogenesis of AMD. This hypothesis was tested by evaluating the effect of H_2_O_2_ on the production of IL-6 by RPE cells. Relevant signal pathways were also studied.

## Methods

### Cell culture

The human retinal pigment epithelial cell line (ARPE-19), was obtained from American Type Culture Collection, Manassas, VA. Cells were cultured in Dulbecco’s modified Eagle’s medium (Gibco, Carlsbad, CA) supplemented with 10% fetal bovine serum (Gibco). Cells were incubated in a humidified 5% CO_2_ atmosphere at 37 °C. After reaching confluence, cells were detached by trypsin-EDTA solution (Gibco), diluted 1:3–1:4, plated for subculture, and passaged routinely at a dilution of 1:3–1:4 every 5–7 days.

A new separate culture of primary human RPE cells was isolated from a donor eye (56 years old) and cultured as previously described [44]. Cells were cultured in Dulbecco’s modified Eagle’s medium with 10% fetal bovine serum. After reaching confluence, cells were subcultured as described previously [44]. Phase-contrast microscopy revealed pigmentation of RPE cells during the primary culture and the first and second subcultures. Cells displayed characteristic epithelial morphology throughout the culture period. The purity of the cell lines was demonstrated by immunocytochemical methods. RPE cells displayed positive staining of cytokeratin, whereas fibroblasts and melanocytes did not [45]. Cells were cultured on chamber slides and immunostained with anti-cytokeratin antibodies (for cytokeratin 6 and 18; Dako, Carpinteria, CA) as described previously [45]. Immunocytochemical study showed that all cells stained positively with anti-cytokeratin antibody, indicating the purity of the RPE cells.

### Effects of hydrogen peroxide on the viability of retinal pigment epithelial cells

The effects of H_2_O_2_ on the viability of RPE cells were studied with a 3-(4,5-dimethylthiazol-2-yl)-2,5 diphenyltetrazolium bromide (MTT) test. Briefly, RPE cells were plated in 96-well plates at a density of 5x10^3^ cells per well. After incubation for 24 h, H_2_O_2_ (Sigma, St. Louis, MO) was added to the wells at various final concentrations (10, 30, 60, 100, and 300 µM) and cultured for 24 h. Then, 50 µl of MTT (1 mg/ml, Sigma) was added to each well and incubated for 4 h. The medium was withdrawn and 100 µl of dimethyl sulfoxide (Sigma) was added to each well. The optical density was read at 540 nm using a microplate reader (Multiskan EX, Thermo, Vantaa, Finland). Cells cultured without H_2_O_2_ were used as the controls. All groups were tested in triplicate.

### Hydrogen peroxide treatment and interleukin-6 protein measurement

RPE cells were plated into 24-well plates at a density of 1×10^5^ cells per well. After 24 h incubation, the medium was withdrawn, and cells were washed with Hanks’ balanced saline solution (Gibco). Fresh medium with different concentrations of H_2_O_2_ (0, 10, 30, 60, and 100 µM) was added to the culture. After 24 h, the conditioned culture medium was collected and centrifuged for 5 min to remove suspended cells. Supernatants were collected and stored at −70 °C until analysis. In the time-dependent study, cells were cultured with H_2_O_2_ (100 µM) for 2, 6, 12, and 24 h and then collected as described above.

The amount of IL-6 protein in the conditioned medium was determined using the human IL-6 Quantikine enzyme-linked immunosorbent assay (ELISA) kit (R&D Systems, Minneapolis, MN) according to the manufacturer’s instructions. Supernatants of condition media were added to 96 well plate precoated with monoclonal antibody against IL-6 and incubated 2 h at room temperature. Conditioned media were aspirated and wells were washed with Wash Buffer four times. Antibody against IL-6 conjugated to horseradish peroxidase was added to the well and incubated 2 h at room temperature. Wells were aspirated and washed with Wash Buffer four times. Color Reagent A (H_2_O_2_) and B (tetramethylbenzidine) were added and incubated for 20 min at room temperature in darkness. Stop solution (sulfuric acid) was added. IL-6 standard at various concentrations was tested simultaneously. Optical density was read by using a microplate reader (Multiskan EX, Thermo, Vantaa, Finland) at 450 nm. The amount of IL-6 (pg/ml) was calculated from the standard curve. The sensitivity of this kit was 0.7 pg/ml. All tests were performed in triplicate.

### Hydrogen peroxide treatment and interleukin-6 mRNA measurement

RPE cells were plated into 6-well plates at a density of 8×10^5^ cells per well. After 24 h incubation, the medium was changed, and H_2_O_2_ was added to the medium at a final concentration at 100 µM. After 2, 4, and 6 h, cells were washed with cold phosphate-buffered saline (PBS; pH 7.4, with components: potassium phosphate monobasic, 0.001 M; sodium chloride, 0.155 M and sodium phosphate dibasic, 0.003 M) three times and then scraped from the well. After centrifugation at 400 xg for 5 min at 4 °C, the cell pellets were collected and stored at −70 °C until analysis. In the dose-dependent study, H_2_O_2_ was added to the medium at various concentrations (0, 10, 30, and 100 µM). Cells were collected and centrifuged 4 h later and stored at −70 °C.

Total RNA was isolated using the Qiagen RNeasy mini kit following the manufacturer’s instructions. The purified RNA was reverse-transcribed to single-stranded cDNA using the SuperScript® First-Strand Synthesis System (Cat. No.11904–018; Invitrogen, Carlsbad, CA). Primers for *IL-6* and *GAPDH* were designed using the Primer3 program as previously described [46,47] (Table 1). Real-time PCR was performed in triplicate for each RNA sample, using the SYBR® Green PCR Master Mix (Cat. No.4309155, Applied Biosystems, Carlsbad, CA) and primers for *IL-6*, *GAPDH*, and beta-actin (*ACTB*). The PCR was cycled in a 7900HT thermal cycler (Applied Biosystems) under the following conditions: 95 °C for 10 min, and 40 cycles of 95 °C for 10 s, 60 °C for 20 s, and 72 °C for 30 s. Relative transcript quantities were determined using Sequence Detection Software (Applied Biosystems). *GAPDH* and *ACTB* were used as references. All of these triplicate tests were assays for three different specimens.

**Table 1:** Primers for *IL-6* and *GAPDH*.

**Gene**	**Accession number**	**Primers (3′-5′)**	**Product**
*IL-6*	NM_000600	F: AGTGAGGAACAAGCCAGAGC	97 bp
		R: CAGGGGTGGTTATTGCATCT	
*GAPDH*	M33197	F: CGACCACTTTGTCAAGCTCA	112 bp
		R: GGTGGTCCAGGGGTCTTACT	

### Hydrogen peroxide treatment and measurement of phosphorylated ERK, JNK, and p38 MAPK

RPE cells were plated into 6-well plates at a density of 1×10^6^ cells per well. After 24 h incubation, the medium was changed, and H_2_O_2_ at a final concentration of 100 µM was added to the medium. After 30 min, the culture medium was withdrawn. Cells were washed with cold PBS three times and then scraped from the well. After cell counting and centrifugation at 400 xg for 5 min at 4 °C, the cell pellets were collected. Cells were lysed using Cell Extraction Buffer (BioSource, Camarillo, CA) with Protease Inhibitor Cocktail (Sigma) and phenylmethylsulfonyl fluoride (BioSource), incubated on ice for 30 min, and vortexed for 30 s. The lysates were centrifuged at 17,500 xg for 10 min at 4 °C. The supernatants were stored at −70 °C until analysis.

The amount of phosphorylated p38 mitogen-activated protein kinases (MAPK), extracellular-signal-regulated kinase (ERK), and c-Jun N-terminal kinases (JNK) in cell lysates were measured using ELISA kits (p38 MAPK kit, #KHO0071; ERK1/2 kit, #KHO0091; and JNK1/2 kit, #KHO0131; Invitrogen, Camarillo, CA) according to the manufacturer’s instructions. The levels of phosphorylated p38 MAPK, ERK, and JNK were calculated using the standard curve and expressed as units/ml (U/ml). One Unit of p38 MAPK is equivalent to the amount of p38 MAPK derived from 40 pg of p38 MAPK which was phosphorylated by MKK6. One Unit of ERK1/2 is equivalent to the amount of ERK1/2 derived from 40 pg of ERK1/2 which was phosphorylated by MEK1. One Unit of JNK1/2 is derived from 50 pg of phosphorylated JNK1 or JNK2 (instructions of p38 MAPK, ERK1/2, and JNK1/2 ELISA kits). The sensitivity of these kits was 0.8 U/ml. All tests were performed in triplicate.

### H_2_O_2_ treatment and measurement nuclear factor-kappaB (NFκB) in nuclear extracts

RPE cells were plated into 6-well plates at a density of 1×10^6^ cells per well. After 24 h incubation, the medium was changed, and H_2_O_2_ at a final concentration of 100 µM was added to the medium. After 30 min, the culture medium was withdrawn. Cells were washed with cold PBS and then scraped from the well. Cells were treated with hypotonic buffer (BioSource) and centrifuged. The pellet (nuclear fraction) was collected and treated with Cell Extraction Buffer (BioSource), vortexed, centrifuged, and the supernatants (nuclear extracts) were stored at −70 °C until analysis.

The amount of nuclear factor-kappaB (NF-κB) in cell nuclear extracts was measured by using NF-κB ELISA kits (#KHO0371, Invitrogen) according to the manufacturer’s instructions. The levels of NF-κB in nuclear extracts were calculated using the standard curve and expressed as pg/ml. The sensitivity of these kits was <50 pg/ml. All of these triplicate tests were assays for three different specimens.

### Effects of MAPK and NF-κB inhibitors on H_2_O_2_-induced release of IL-6 by RPE cells

RPE cells were plated into 24-well plates at a density of 1×10^5^ cells per well. After 24 h incubation, the medium was changed, and various MAPK or NF-κB inhibitors were added to the medium separately, including 5 µM BAY11–7082 (NF-κB inhibitor), 10 µM UO1026 (ERK inhibitor), 10 µM SP600125 (JNK inhibitor), and 10 µM SB203580 (p38 MAPK inhibitor), all from Calbiochem, San Diego, CA. After 30 min, H_2_O_2_ was added to the medium at a final concentration of 100 µM. After 24 h incubation, the conditioned medium was collected and centrifuged. The amount of IL-6 in the supernatant was determined using the human IL-6 Quantikine ELISA kit, as described above. Cells cultured with medium without H_2_O_2_ were used as negative controls and cells cultured with H_2_O_2_ but without inhibitors were used as the positive controls. All tests were performed in triplicate.

### Effects of p38 inhibitors on H_2_O_2_-induced increase of NF-κB in nuclear extracts

RPE cells were plated into 6-well plates at a density of 1×10^6^ cells per well. After 24 h incubation, the medium was changed, and SB203580 (p38 MAPK inhibitor) at a final concentration of 10 µM was added. After 30 min, H_2_O_2_ at a final concentration of 100 µM was added to the medium as described above. After another 30 min, the culture medium was withdrawn. Cells were collected and the amount of NF-κB in nuclear extracts was measured by using the NF-κB ELISA kit, as described above. Cells cultured without H_2_O_2_ and with H_2_O_2_ but without SB203580 were used as negative and positive controls, respectively. Tests were performed in triplicate.

### Statistical analysis

Statistical significances of difference throughout this study were analyzed with one way ANOVA with a Student-Newman-Kuels post hoc test. A difference at p<0.05 and p<0.01 was considered to be significant and very significant, respectively.

## Results

### Effects of H_2_O_2_ on the viability of RPE cells

In RPE cells (both ARPE-19 and the primary cultures) cultured with H_2_O_2_ at 10–100 µM, the MTT assay showed no significant difference as compared with cells cultured without H_2_O_2_, indicating that H_2_O_2_ at 10–100 µM did not affect the viability of RPE cells. Cell viability in these two cell lines decreased only by 300 μM H_2_O_2_. Cell viability in ARPE-19 and the primary cultures cultured with 300 μM H_2_O_2_ was 79.6±6% (mean±standard deviation) and 81±5% of the controls (p=0.0218 and 0.0190), respectively. Therefore, H_2_O_2_ at 10–100 µM was used to study the effects of subtoxic levels of H_2_O_2_ on the expression and release of IL-6 from RPE cells.

### Effects of H_2_O_2_ on the release of IL-6 by RPE cells

**Figure 1:**
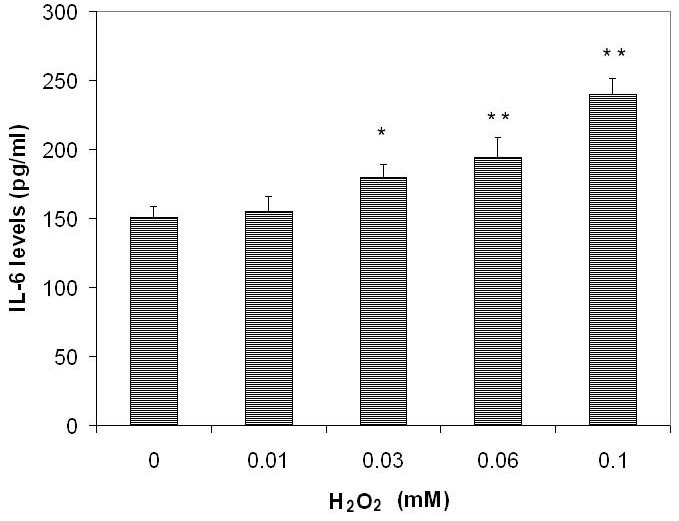
The dose-dependent effects of subtoxic levels of hydrogen peroxide on release of interleukin-6 by retinal pigment epithelium cells. Retinal pigment epithelium (RPE) cells (ARPE-19) were plated into 24-well plates. After 24 h incubation, hydrogen peroxide (H_2_O_2_, at 0, 10, 30, 60, and 100 µM) was added to the culture and incubated for 24 h. Conditioned culture medium was collected and the amount of interleukin-6 (IL-6) protein in the conditioned medium was determined using the human IL-6 Quantikine enzyme-linked immunosorbent assay (ELISA) kit. IL-6 levels in conditioned culture medium were expressed as pg/ml (mean±standard deviation in triplicate tests). *0.01< p<0.05, **p<0.01, compared with the controls (cells cultured without H_2_O_2_).

H_2_O_2_ increased the level of IL-6 protein in the culture medium of ARPE-19 cells in a dose-dependent manner (Figure 1). IL-6 level in conditioned medium from cells cultured without H_2_O_2_ was 150.7±7.9 pg/ml (mean± standard deviation [SD]). IL-6 levels in conditioned medium from cells cultured with H_2_O_2_ at 10, 30, 60, and 100 µM were 1.05-fold, 1.22-fold, 1.52-fold, and 1.82-fold, respectively, of those of the controls (Figure 1). IL-6 levels were not different comparing 0 and 10 μM H_2_O_2_ (p=0.5866). Compared 30 μM H_2_O_2_ there was a difference (p=0.0169) and a larger difference 60 and 100 μM H_2_O_2_ (p=0.0097 and 0.0005, respectively).

**Figure 2:**
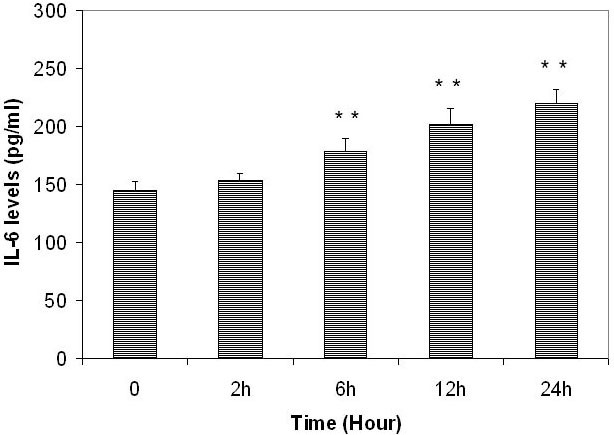
The time-dependent effects of subtoxic levels of hydrogen peroxide on release of interleukin-6 by retinal pigment epithelium cells. Retinal pigment epithelium (RPE) cells (ARPE-19) were plated into 24-well plates. After 24 h incubation, 100 µM hydrogen peroxide (H_2_O_2_) was added to the culture and incubated for 2, 6, 12, and 24 h. Conditioned culture medium was collected and the amount of interleukin-6 (IL-6) protein in the conditioned medium was determined by using the human IL-6 Quantikine enzyme-linked immunosorbent assay (ELISA) kit. IL-6 levels in conditioned culture medium were expressed as pg/ml (mean±standard deviation in triplicate tests). *0.01< p<0.05, **p<0.01, compared with the controls (cells cultured without H_2_O_2_).

The H_2_O_2_-induced increase of release of IL-6 by ARPE-19 cells was also time dependent (Figure 2). The IL-6 level in conditioned medium from cells cultured without H_2_O_2_ was 144.2±7.8 pg/ml. IL-6 levels in conditioned medium from cells cultured with H_2_O_2_ (100 µM) for 2, 6, 12, and 24 h were 1.12-fold, 1.26-fold, 1.31-fold, and 1.82-fold of the controls, respectively (Figure 2). IL-6 levels were not different comparing 0 and 100 μM H_2_O_2_ treated for 2 h (p=0.1129). Compared after 6h there was a difference (p=0.0269) and a larger difference after 12 and 24 h (p=0.0091 and 0.0009, respectively).

H_2_O_2_ also induced increased release of IL-6 by human RPE cells isolated from a 56-year-old donor in a dose- and time-dependent manner. The IL-6 level in conditioned medium from cells cultured without H_2_O_2_ was 132.1±9.6 pg/ml. IL-6 levels were not different comparing 0 and 10 μM H_2_O_2_ (p=0.6024). Compared 30 and 60 μM H_2_O_2_ there was a difference (p=0.0456 and 0.0110, respectively) and a larger difference 100 μM H_2_O_2_ (p=0.0005). IL-6 levels were not different comparing 0 and 100 μM H_2_O_2_ treated for 2 h (p=0.1129). Compared after 6 h there was a difference (p=0.0269) and a larger difference after 12 and 24 h (p=0.0091 and 0.0009, respectively).

### Effects of H_2_O_2_ on *IL-6* mRNA level in RPE cells

The real-time PCR experiment demonstrated that H_2_O_2_ upregulated *IL-6* mRNA levels in RPE cells. In the time-dependent study, the expression of IL-6 in H_2_O_2_-treated cells (100 µM) increased to 1.57±0.04, 1.97±0.09, and 1.95±0.07 fold of the controls after 2, 4, and 6 h treatments, respectively. In the dose-dependent study, the *IL-6* mRNA level in H_2_O_2_-treated cells (10, 30, and 100 µM) increased to 1.21±0.04, 1.36±0.08, and 1.97±0.09 fold of the controls, respectively, after 4 h treatment. The difference in the *IL-6* mRNA levels between the H_2_O_2_-treated cells (100 µM) and the controls was significant (p=0.0189).

### Effects of H_2_O_2_ on phosphorylated ERK, JNK, and p38 MAPK level in RPE cells

**Figure 3:**
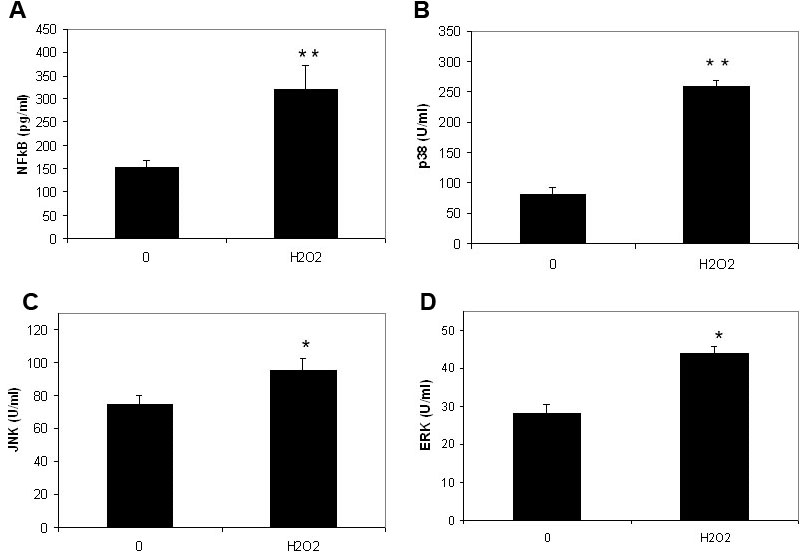
The effects of subtoxic levels of hydrogen peroxide on nuclear factor factor-kappaB in nuclear extracts and phosphorylated extracellular signal-regulated kinases, c-Jun N-terminal kinase and p38 mitogen-activated protein kinase. Retinal pigment epithelium (RPE) cells (ARPE-19) were plated into 24-well plates. After 24 h incubation, 100 µm hydrogen peroxide (H_2_O_2_) was added to the medium. Cells were collected 30 min later. The nuclear factor factor-kappaB (NF-κB) levels in nuclear extracts (**A**) and phosphorylated p38 mitogen-activated protein kinase (p38; **B**), c-Jun N-terminal kinase (JNK; **C**) and extracellular signal-regulated kinases (ERK; **D**) in cell lysates were measured using the relevant NF-κB enzyme-linked immunosorbent assay (ELISA) kit and phosphorylated MAPK ELISA kits (Biosource). The levels of NF-κB in nuclear extracts were expressed as pg/ml and phosphorylated p38, ERK and JNK in cell lysates were expressed as U/ml (mean±standard deviation in triplicate tests). *0.01< p<0.05, **p<0.01, compared with the controls (cells cultured without H_2_O_2_).

H_2_O_2_ treatment (100 µM with 30 min incubation) increased phosphorylated ERK, JNK, and p38 MAPK levels in ARPE-19 cells (Figure 3). The level of phosphorylated p38 MAPK in cells cultured without H_2_O_2_ was 81.2±11.0 U/ml and in H_2_O_2_ treated cells this increased to 3.19 fold of the controls (Figure 3B). The difference in phosphorylated p38 MAPK levels between cells treated with and without H_2_O_2_ was statistically very significant (p=0.00003). The increase of phosphorylated ERK and JNK levels in H_2_O_2_ treated cells was much less than that of p38. The levels of phosphorylated ERK and JNK in cells cultured without H_2_O_2_ were 28.2±2.3 and 74.5±5.7 U/ml, respectively. The level of phosphorylated ERK and JNK in H_2_O_2_-treated cells only increased to 1.56 and 1.28 fold of the controls, respectively (Figure 3C,D). The difference in phosphorylated ERK and JNK levels between cells treated with and without H_2_O_2_ was statistically significant (p=0.0315 and 0.0177, respectively).

### Effect of H_2_O_2_ on NF-κB levels in nuclear extracts from RPE cells

H_2_O_2_ treatment (100 µM with 30 min incubation) increased NF-κB levels in nuclear extracts from ARPE-19 cells (Figure 3A). The levels of NF-κB in nuclear extracts in cells cultured with and without H_2_O_2_ were 318.7±54.4 U/ml and 152.3±15.6 U/ml, respectively (Figure 3A). The difference of NF-κB levels between cells treated with and without H_2_O_2_ was statistically very significant (p=0.0012).

### Effects of MAPK and NF-κB inhibitors on H_2_O_2_-induced increase of IL-6 release by RPE cells

**Figure 4:**
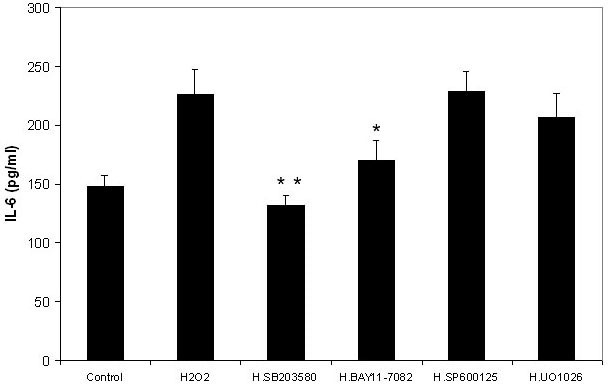
Effects of nuclear factor factor-kappaB and mitogen-activated protein kinase inhibitors on the hydrogen peroxide-induced production of interleukin-6 by retinal pigment epithelium cells. Retinal pigment epithelium (RPE) cells (ARPE-19) were plated into 24-well plates. After 24 h incubation, various mitogen-activated protein kinase (MAPK) and nuclear factor-kappaB (NF-κB) inhibitors were added to the medium separately, including BAY11–7082 (NF-κB inhibitor), UO1026 (extracellular signal-regulated kinases inhibitor), SP600125 (c-Jun N-terminal kinase inhibitor) and SB203580 (p38 MAPK inhibitor, all from Calbiochem) at a final concentration of 10 µM with the exception of BAY11–7082 (5 µM). After 30 min, 100 µM hydrogen peroxide (H_2_O_2_) was added to the medium. Cells cultured without H_2_O_2_ were used as negative controls. Cells cultured with H_2_O_2_ but without inhibitors were used as positive controls. After 24 h incubation, the culture medium was collected and the IL-6 levels were measured with the human IL-6 Quantikine ELISA kit and expressed as pg/ml (mean±standard deviation in triplicate tests). *0.01< p<0.05, **p<0.01, compared with the positive controls (cells cultured with H_2_O_2_ but without inhibitors).

Treatment of ARPE-19 cells with SB203580 (p38 MAPK inhibitor) 30 min before the H_2_O_2_ addition completely abolished H_2_O_2_-induced release of IL-6 (Figure 4). The difference in the amount of IL-6 in medium between cells treated with and without SB203580 (positive control) was very significant (p=0.0022). The level of IL-6 in medium from SB203580 and H_2_O_2_ treated cells was similar to that of negative controls (cells cultured without H_2_O_2,_ p=0.0947). These results indicated that the p38 inhibitor (SB 203580) completely abolished the H_2_O_2_-induced increase of release of IL-6 by RPE cells.

Pretreatment of ARPE-19 cells with BAY11–7082 (NF-κB inhibitor) 30 min before the H_2_O_2_ addition decreased 72% of H_2_O_2_-induced release of IL-6 (Figure 4). The difference in the amount of IL-6 in medium between ARPE-19 cells treated with and without BAY11–7082 (positive control) was significant (p=0.0232). The levels of IL-6 in medium from BAY11–7082 and H_2_O_2_ treated cells was slightly greater than that of negative controls (cells cultured without H_2_O_2_); however, there was no significant difference between these two groups (p=0.1155), indicating that the NF-κB inhibitor inhibited the H_2_O_2_-induced increase of the release of IL-6 by RPE cells.

Treatment with UO1026 (ERK inhibitor) and SP600125 (JNK inhibitor) 30 min before the H_2_O_2_ addition only showed a minor influence on the H_2_O_2_-induced release of IL-6 by ARPE-19 cells (Figure 4). The IL-6 levels in conditioned media from cells cultured with UO1026 or SP600125 were similar to that of cells treated with H_2_O_2_ (positive control). There was no significant difference in the release of IL-6 between cells treated with or without UO1026 (p=0.4202) and cells treated with or without SP600125 (p=0.8917).

### Effects of p38 inhibitor on the H_2_O_2_-induced increase of NF-κB in nuclear extracts

NF-κB levels in nuclear extracts from cells cultured without H_2_O_2_, with H_2_O_2,_ and with H_2_O_2_ and p38 inhibitor were 146±13.5, 323±21.9, and 152.7±7.8 pg/ml, respectively. The difference of NF-κB levels between cells cultured without H_2_O_2_ and with H_2_O_2_ and p38 inhibitor was non-significant (p=0.6208) and the difference between cells cultured with H_2_O_2_ and H_2_O_2_ and p38 inhibitor was very significant (p=0.002). This indicated that the increase of NF-κB levels in nuclear extracts induced by H_2_O_2_ could be completely abolished by the p38 inhibitor.

## Discussion

There has been increasing evidence suggesting a role for inflammation and aberrant complement activation in the pathogenesis of AMD [10-26]. Chronic inflammatory infiltrates have been demonstrated in the choroid and excised choroidal neovascular membranes (CNV) from AMD [5,10-12,18,19]. Analyses of drusen composition in AMD patients and animal models have identified various inflammatory proteins (e.g., acute phase proteins, cytokines, etc.), immunoglobulin, and components of the complement cascade [5,10-12,18,19]. Complement factor H (CFH), a key inhibitor of the alternative pathway of complement activation, can bind and inactivate harmful complement components and prevent damage of intact host cells. CFH gene polymorphism has been reported to be an important risk factor for AMD. Dysfunction of the complement system may result in local inflammation, autoimmune reactions, and tissue damage, and may play an important role in the etiology of AMD [11,12,15,17,20].

There are several possibilities linking ROS and inflammatory processes in AMD. ROS may cause damage of RPE cells and result in cell debris deposits between the RPE and Bruch’s membrane, which may in turn induce an inflammatory reaction [5,10-12]. Prolonged phagocytosis of oxidized photoreceptor outer segments may reduce the production of CFH, which could lead to overactivation of complement cascades [21]. Immunization with an oxidant product of docosahexaenoic acid in the retina produced AMD-like lesions in mice [22].

IL-6 is the key factor for the stimulation of immune reactions, inflammatory processes, and the occurrence of autoimmune diseases [27-30]. Increased systemic or local IL-6 levels have been observed in various autoimmune diseases [27-33]. IL-6–deficient mice are resistant to various experimentally induced autoimmune diseases [28,29].

IL-6 also plays a role in inflammatory processes. At the beginning of inflammation, IL-6 mediates the acute phase response. It stimulates the production of various acute phase proteins (C-reactive protein, α-antitrypsin, α-antichymotrypsin, fibrinogen, etc., most of which are present in drusen) by cells to cause a systemic reaction and local inflammation [30]. IL-6 synergistically induces production of VEGF by cells with IL-1 or TNF-α [48]. IL-6 decreases the synthesis of CFH by RPE cells, which may result in the overactivation of complement cascades, inflammatory processes, and autoimmune reactions [21].

Seddon et al. [14] reported that plasma IL-6 levels were related to the progression of AMD. In a population-based multi-ethnic study involving 5,887 persons, Klein et al. reported that increased IL-6 plasma levels are definitely associated with geographic atrophy (odds ratio: 2.06, 95% confidence interval, 1.21–3.49) [13]. Aqueous humor IL-6 level is correlated with the size of CNV in activate CNV patients, indicating that IL-6 level may be related to the progression of CNV [49].

ROS were first considered only as cytotoxic substances that induce cell death at high concentrations. In the past few years, however, they have gained attention as potential signaling molecules at subtoxic levels. ROS at physiologic concentrations are able to modulate various cell functions [6-9]. Actually, in the pathological process of AMD, death of RPE cells (geographic atrophy) occurs only at a later stage [50]. Therefore, studying the effect of subtoxic levels of ROS on the function of RPE cells is important for the elucidation of early pathologic processes in AMD.

H_2_O_2_ is a relatively weak oxidant, but in the presence of metal catalysts it can be converted to the reactive hydroxyl radical, which is cytotoxic and can cause cell death. H_2_O_2_ is the most stable ROS, so that it is present in tissues with a relatively long half-life. H_2_O_2_ is soluble in both lipid and aqueous media, and therefore it easily diffuses in and out of the cell to reach targets. Subtoxic levels of H_2_O_2_ can influence signaling pathways and induce changes in various cell functions [6-9]. H_2_O_2_ has been used as a model of exogenous oxidative stress for testing both cytotoxic and nontoxic effects on ocular cells [50-56]. The RPE is continuously exposed to high oxygen fluxes because of its location between the photoreceptors and choroid (where there is a high oxygen partial pressure from the underlying choriocapillaris), and has a high metabolic activity. A high level of oxidative stress occurs in RPE cells, causing higher levels of H_2_O_2_. During photoreceptor phagocytosis, RPE cells generate H_2_O_2_, which can promote endogenous oxidative stress [3,5,51].

Recently, it has been reported that subtoxic levels of H_2_O_2_ stimulate the expression and release of IL-6 in fibroblasts, mast cells, skeletal myocytes, and trabecular cells [39-43], but not in glioma cells or bronchial epithelial cells [57,58]. This indicates that this effect is highly cell type–specific. RPE cells constitutively express and release IL-6 [34-38]. However, the effects of H_2_O_2_ on the expression and release of IL-6 by RPE cells was unknown in previous studies.

In the present study, subtoxic levels of H_2_O_2_ (30–100 µM) stimulated the expression and secretion of IL-6 by human RPE cells in vitro. These levels are compatible with that observed during phagocytosis of the outer segments of photoreceptors by RPE cells. It has been reported that H_2_O_2_ generated by RPE cells during phagocytosis and the degradation of photoreceptor outer segments can reach 29–40 µM after 4 h exposure [51].

MAPK signaling pathways represent one of the most important pathways influenced by H_2_O_2_ [6]. This family mainly consists of ERK1/2, JNK, and p38 kinase pathways. In previous studies, H_2_O_2_ stimulated the production of IL-6 in rat fibroblasts and myocytes through the activation of p38, JNK, or ERK signaling pathways, mainly through the p38 pathway [39,42].

In the present study, H_2_O_2_ at subtoxic levels increased phosphorylated p38 MAPK levels in RPE cells. A p38 inhibitor could completely abolish the H_2_O_2_-induced increase of IL-6 release by RPE cells, indicating that the stimulation of IL-6 production in RPE cells by H_2_O_2_ mainly occurs through the activation of the p38 signaling pathway, which is consistent with previous studies on other cell types [39,42].

In this study, H_2_O_2_ induced an increase of NF-κB levels in nuclear extracts. An NF-κB inhibitor decreased the H_2_O_2_-induced increase of IL-6 release by RPE cells, indicating that NF-κB is involved in the H_2_O_2_-induced expression of IL-6. A p38 inhibitor completely abolished the H_2_O_2_-induced increase of NF-κB levels in nuclear extracts, indicating that H_2_O_2_-induced NF-κB activation is dependent on p38 activation. H_2_O_2_ activates the p38 signal pathway, which leads to activation and translocation of NF-κB to the nuclei and in turn leads to an increased release of IL-6. This is consistent with the results of previous studies, which showed that NF-κB activation in various cell lines via different stimulators is blocked by the p38 inhibitor, and that NF-κB activation is dependent on p38 phosphorylation [59-64].

The present study found that H_2_O_2_ stimulated the production of IL-6, a key factor in the modulation of immune responses, inflammatory processes, and the occurrence of autoimmune diseases, which recently has been documented to be increased in AMD. This could be a molecular linkage for the oxidative stress and inflammatory/autoimmune reactions in AMD, and may provide a novel target for the treatment of AMD.

Recently, progress on the understanding of IL-6 pathobiology and its role in autoimmune reactions has led to the improvement in the treatment of autoimmune diseases using novel anti-IL-6 drugs [27,28,65,66]. The future perhaps lies in the development of orally active small molecules that inhibit specific inflammatory signaling pathways, e.g., the inhibitor of p38 and its upstream effectors have been proven to be effective in the treatment of autoimmune arthritis in a rat model and have been recently tested in clinical trials [67-70]. These novel therapeutic strategies may have a beneficial effect in the prevention and control of AMD and warrant further investigation.
